# Common Functional Brain States Encode both Perceived Emotion and the Psychophysiological Response to Affective Stimuli

**DOI:** 10.1038/s41598-018-33621-6

**Published:** 2018-10-18

**Authors:** Keith A. Bush, Anthony Privratsky, Jonathan Gardner, Melissa J. Zielinski, Clinton D. Kilts

**Affiliations:** 1 Brain Imaging Research Center, University of Arkansas for Medical Sciences, 4301 W. Markham St., Little Rock, AR 72205-7199 USA; 2 College of Medicine, University of Arkansas for Medical Sciences, 4301 W. Markham St., Little Rock, AR 72205-7199 USA

## Abstract

Multivariate pattern analysis (MVPA) of functional magnetic resonance imaging (fMRI) data has critically advanced the neuroanatomical understanding of affect processing in the human brain. Central to these advancements is the brain state, a temporally-succinct fMRI-derived pattern of neural activation, which serves as a processing unit. Establishing the brain state’s central role in affect processing, however, requires that it predicts multiple independent measures of affect. We employed MVPA-based regression to predict the valence and arousal properties of visual stimuli sampled from the International Affective Picture System (IAPS) along with the corollary skin conductance response (SCR) for demographically diverse healthy human participants (n = 19). We found that brain states significantly predicted the normative valence and arousal scores of the stimuli as well as the attendant individual SCRs. In contrast, SCRs significantly predicted arousal only. The prediction effect size of the brain state was more than three times greater than that of SCR. Moreover, neuroanatomical analysis of the regression parameters found remarkable agreement with regions long-established by fMRI univariate analyses in the emotion processing literature. Finally, geometric analysis of these parameters also found that the neuroanatomical encodings of valence and arousal are orthogonal as originally posited by the circumplex model of dimensional emotion.

## Introduction

Multivariate pattern analysis (MVPA) of functional magnetic resonance imaging (fMRI) data has critically informed our mechanistic understanding of affect and emotion processing. Central to these MVPA-based advancements is the brain state^1–7^, defined in this work as a temporally-succinct fMRI-derived pattern of neural activation, which serves as a unit of cognitive processing. Brain states, conforming to ours and similar definitions, have repeatedly demonstrated the ability to predict affective information via MVPA^8–16^.

Within the subset of experiments specifically focused on decoding affective information from complex visual imagery, MVPA has been deployed to significantly classify brain states across both the discrete^17^ and dimensional^7,18–20^ models of emotion processing. MVPA of brain states has significantly predicted the subjective experience of emotional valence (the degree of pleasantness-unpleasantness) measured via both self-report^19^ and normative affective stimulus rating scores^7,18–20^. These classifiers also predicted normative affective stimulus rating scores of arousal, i.e., the degree of intensity^7,18,19^, which are theorized to exist orthogonally to those of valence in the circumplex model of dimensional emotion^21^. Combined, these findings suggest that brain states represent a functional neuroanatomical analogue to self-reported experiences of emotion.

Contrary to the assumptions inherent within past classification studies, however, affect processing exhibits a property of degree, i.e., affective stimuli induce graded experiential responses. Past studies of MVPA-based classification of fMRI-derived brain states ignored this property and instead modeled affect as having binary properties (positive/negative valence and high/low arousal). Yet, degree is fundamental to the dimensional model of emotion; it is the means by which the continuously-valued dimensions of valence and arousal (and possibly dominance and others) independently assign unique emotional experiences to potentially similar but novel stimuli or affective cognitions. Past MVPA-based classification studies also ignored well-established psychophysiological measures of affective state as a means to independently measure the validity of affective brain states. For example, the skin conductance response (SCR) reflects activation of the sympathetic autonomic nervous system, i.e., arousal^22^, a broad preparatory response (the instantiation of alertness and readiness to act) that is associated with certain cognitive processes or recruited in response to stimuli^23^.

Thus, while there exist sufficient data to support affective brain states as a promising component within the long-term search for a mechanistic explanation of affect processing, lingering gaps in the evidence of their validity are problematic for the establishment of fMRI-derived brain states as the central unit of affect processing. Therefore, the primary goal of this work is to test a conceptual model of the brain state as the central unit of affect processing within the dimensional model of emotion by (1) predicting multiple, continuously-valued dimensions of affect from brain states defined by a single measurement modality, the fMRI-derived blood oxygen-level dependent (BOLD) signal, while (2) simultaneously predicting an independent measure of stimulus-driven affect perception based on well-studied psychophysiological response. (3) Verifying the anatomical consistency of the derived brain states with respect to functional regions long-established in the fMRI-based emotion processing literature across multiple paradigms as well as (4) verifying the anatomical orthogonality of valence and arousal processing performed by brain states that is imposed by the circumplex model of dimensional emotion.

**Figure 1:**
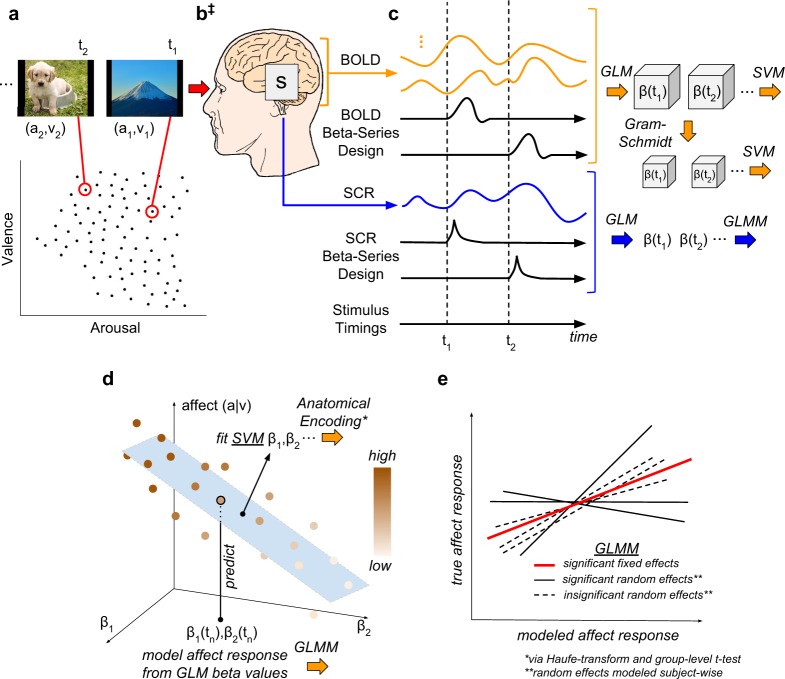
Methodological Overview and Conceptual Model. **(a)** Experiment Design: Ninety images were sampled from the International Affective Picture System (IAPS) to form a subset that maximally spanned the affective properties of valence (v) and arousal (**a**). (**b**) Signal Acquisition: Image stimuli were presented for 2 s interleaved with random inter-trial intervals [2–6 s]; fMRI measurements of the blood oxygen level dependent (BOLD) response were recorded concurrently with the skin conductance response (SCR). (**‡**) Conceptual Model: We hypothesize that brain states, s, simultaneously encode the dimensional affective properties of their image stimuli as well as the attendant psychophysiological responses. **(c)** Brain and Physiological State Estimation: fMRI signals were preprocessed to remove noise and motion artifacts and segmented to remove all voxels except gray matter (GM); SCR signals were preprocessed to remove noise and tonic signal components; neural activation patterns were extracted for each stimulus according to the beta-series method; and, dimensionally reduced. **(d)** Prediction of Affective Signals: Intra-subject cross-validated linear support vector machine (SVM) regression was conducted on the beta-series (labeled according to the stimulus from which they were extracted). The figure depicts the regression model labeling the affective property of a novel point. **(e)** Effect Size Estimation: Group-level predictions of affective properties and measurements were conducted via General Linear Mixed-Effects Models (GLMMs) in three tests: (1) the measurements of interest were the normative affective properties of the stimuli (v, a) and the fixed effects were the SCR measurements of affect state (β^SCR^); (2) the measurements of interest were the affective properties (v, a) and the fixed effects were the SVM-predicted properties (~v, ~a); and, (3) the measurements of interest were β^SCR^ and the fixed effects were the SVM-predicted affective responses (~β^SCR^). (*) The individual SVM models of GM-based features were transformed into encoding representations of affect state^24^ and anatomically analyzed group-wise (not pictured). (**) GLMM random effects for slope and intercept were modeled subject-wise. Note, details of the experiment design, preprocessing pipeline, and brain state estimation methodology have been reported previously^7^.

To achieve this goal we conducted analysis of fMRI and SCR measurements that were concurrently recorded during a visual stimulus-based affective perception experiment. This analysis observed the methodology and conceptual model presented in Fig. 1.

## Results

### Brain state is a central unit of affect processing

**Figure 2:**
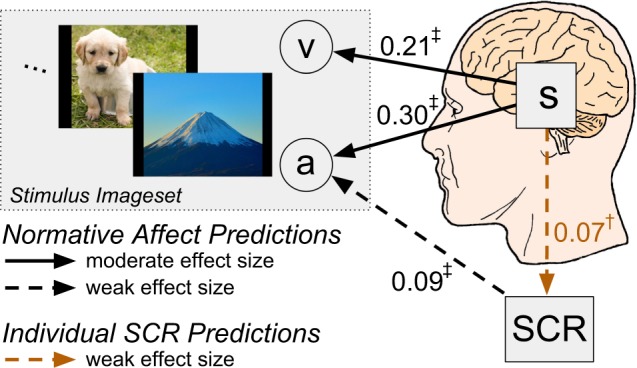
Summary of the primary experimental findings in relation to the proposed conceptual model. Affective properties of the IAPS imageset, valence (v) and arousal (a), brain state (s), and skin conductance responses (SCR) are depicted alongside arrows indicating the direction of significant GLMM-based predictions (^†^p < 0.05, ^‡^p < 0.001, F-test), reported in units of effect size (Pearson’s r).

The primary findings of the GLMM-based analysis of affect encoding are summarized in Fig. 2. Observed SCR significantly predicts the arousal property but not the valence property of affective stimuli (see Supp. Figs S1 and S2). In contrast, affective brain state significantly and simultaneously predicts the arousal property and the valence property of the stimuli (see Supp. Fig. S3). Indeed, brain state predicts arousal with an effect size more than three times greater than that of SCR. Moreover, affective brain states significantly predict observed individual SCRs (see Supp. Fig. S4), supporting its centrality in the processing of affective information.

### SVM hyperplanes recapitulate canonical functional neuroanatomical correlates of affect processing

In order to validate our dimensionality reduction methodology (see Materials and Methods: BOLD Beta-series Dimensionality Reduction), we compared SVM predictions resulting from the two types of affective brain states (see Fig. 1, panel C): full gray matter (GM) beta-series and Gram-Schmidt (GS) dimensionally-reduced beta-series. For all three measures of affect processing explored in this analysis, Gram-Schmidt based SVM predictions significantly predict whole-brain gray matter based SVM predictions (see Supp. Fig. S5): valence (fixed effect: r = 0.93, p < 0.001, F-test); arousal (fixed effect: r = 0.90, p < 0.001, F-test); and, observed SCR (fixed effect: r = 0.88, p < 0.001, F-test). This suggests both that the information content of these states are equally informative of affective information and that the learning architecture’s generalization performance is robust to high-dimensionality imposed by GM features, an established property of the SVM architecture^24^.

**Figure 3:**
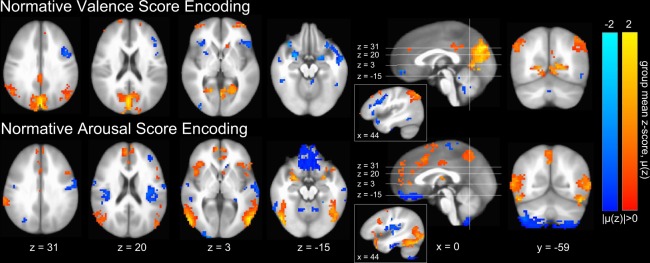
Group-level gray matter mean intra-subject emotion perception encodings^24^ of normative affect properties. Color gradations indicate the group-level mean encoding parameter (red indicating positive valence or high arousal, blue indicating negative valence or low arousal). Only group-level significant parameters (p < 0.05, global permutation test^27^) are depicted (see Materials and Methods: Neuroanatomical Encoding Parameter Significance via Permutation Testing). Image slices are presented in Talairach coordinate space and neurological convention. Maximum mean voxel intensity |μ(z)| = 2.0. Only those clusters having 10 or greater contiguous voxels (NN = 1) are plotted.

We also conducted inter-subject predictions of normative affect scores via group-averaged GM-based intra-subject decoding model predictions (see Materials and Methods: Inter-subject Predictions) and found significant, strong correlations between the predicted and target normative measurements (see Supp. Fig. S6) for both valence (fixed effect: r = 0.61, p < 0.001, F-test) and arousal (fixed effect: r = 0.47, p < 0.001, F-test), which infers strong group similarity among the normal vectors of the underlying model hyperplanes, supporting group-level analysis of the models. Therefore, we exploited the linearity of the SVM model to generate anatomic GM-based voxel-wise encoding models of perceived affect^25,26^ (see Materials and Methods: Construction of Neuroanatomical Encoding Parameters). We then computed group-level mean encoding parameters, respectively, for valence and arousal. Group-level significant parameters (p < 0.05, global permutation test^27^) are depicted graphically in Fig. 3 (see Materials and Methods: Neuroanatomical Encoding Parameter Significance via Permutation Testing).

With respect to the encoding patterns of perceived affective valence and arousal in Fig. 3, our results are in excellent agreement with the neuroanatomical distribution of univariately-identified regions activated in response to both core affective experiences (amygdala, anterior insula [aIns], orbital frontal cortex, striatum, as well as the dorsal [dCC], and to a lesser extent, rostral and middle cingulate cortices) and conceptualization of stimulus content (ventral medial prefrontal cortex [vmPFC], dorsal medial PFC, medial temporal lobe [mTL], hippocampus, and posterior cingulate cortex [pCC])^28^. Indeed, the identified brain structures largely overlap with univariately-identified regions that are activated during discrete emotion induction^29^. We also observe finer grain features of affect processing, e.g., left lateralization of regions (aIns and striatum) that preferentially encode negative affective valence in accordance with recent meta-analysis^30^.

Our encoding models also largely agree with functional neuroanatomical findings from prior MVPA-based neural pattern classifications of emotion processing that have been identified, respectively, for discrete^17^ and dimensional^7,19^ representations of emotion. Indeed, these prior studies reported perception encodings dominated by activations in bi-lateral amygdala, aIns, vmPFC, dCC, pCC, precuneus, and medial occipital cortex (mOC). Non-canonical involvement of precuneus and mOC (with respect to univariately-identified functional neuroanatomy) may reflect the common imagery-based induction methodology shared across these studies.

**Figure 4:**
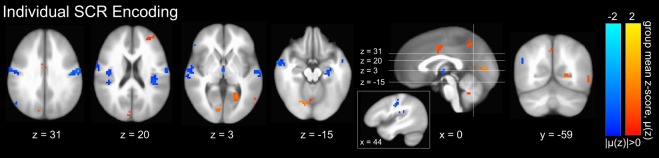
Group-level gray matter mean intra-subject encodings^24^ of individual SCRs. Color gradations indicate the group-level mean activation (red indicating high SCR, blue indicating low SCR). Only group-level significant parameters (p < 0.05, global permutation test^27^) are depicted. Image slices are presented in Talairach coordinate space using the neurological convention. Maximum mean voxel intensity |μ(z)| = 2.0. Only those clusters having 10 or greater contiguous voxels (NN = 1) are plotted.

Similar to our inter-subject normative affect predictions, we generated inter-subject predictions of SCRs based upon group-averaged GM-based intra-subject decoding model predictions and found them to be significantly correlated with observed individual SCR beta activations (fixed effect: r = 0.09, p = 0.002, F-test; see Supp. Fig. S7). This finding suggests the existence of group-level similarity among the normal vectors of the intra-subject models and supports group-level analysis. Therefore, we generated anatomic GM-based voxel-wise encoding models of individual SCRs and computed the group-level mean encoding parameters. Group-level significant parameters (p < 0.05, global permutation test^27^) are depicted in Fig. 4. These parameters suggest that, indeed, SCRs independently detect components of brain states exhibiting properties of perceived arousal, which are highlighted by activations in dCC and precuneus that have been reported both here (see Fig. 3) as well as in other studies pursuing neural correlates of SCR^31^.

### The dimensional property of affective valence is neurally encoded orthogonally to the property of arousal

**Figure 5:**
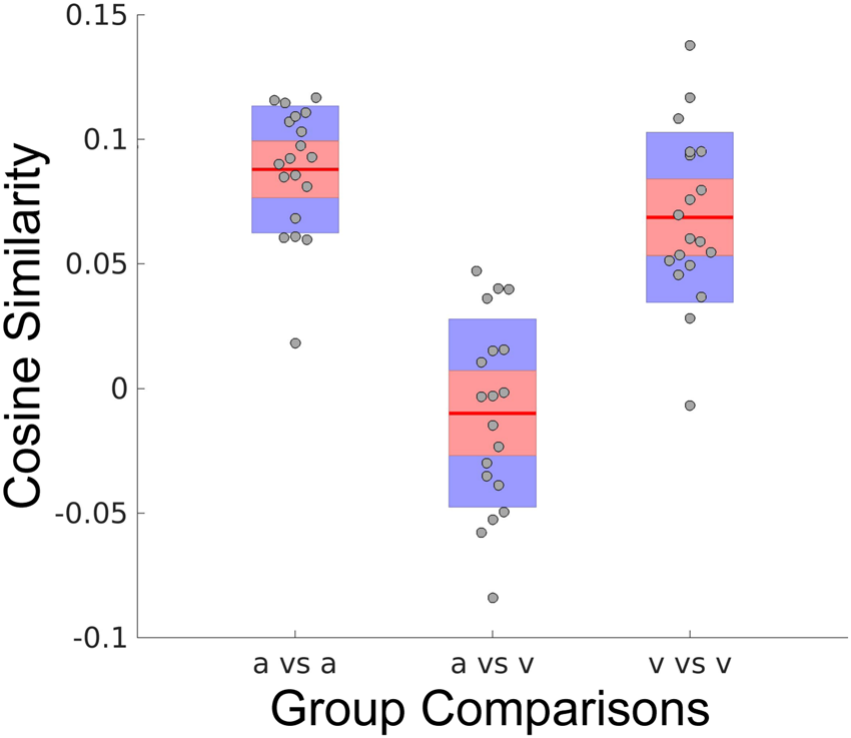
Quantitative group-level comparison of the cosine similarities within and between the neural activation encodings of arousal (a) and valence (v). Similarities are presented as boxplots; gray circle markers depict group-level mean similarity scores for each participant; horizontal red segments indicate the mean distribution values; upper and lower red filled areas represent the 95% confidence intervals of the means; and, blue filled areas represent the distributions’ first standard deviations. Neural encoding similarity is measured according to the cosine of the angle between the vectors describing the compared encoding models of the SVM hyperplanes (see Materials and Methods: Calculation of Cosine Similarity). For each participant, the group-level similarity is formed from the group average between the participant’s hyperplane and the comparison hyperplanes (excluding self-similarity).

Within the circumplex model of dimensional affect^21^, valence and arousal are proposed to exist as orthogonal properties. This is an intuitive conceptual model and obtains support from the inability of a single physiological measurement modality to significantly predict both properties simultaneously. Here we exploit the neural activation encodings (see Fig. 3) of our SVM hyperplanes to compute group-level distributions of the vector angles that exist between encodings of the affective properties of valence and arousal, depicted in Fig. 5. Group-level encodings are significantly similar within the affect properties of arousal (p < 0.001, 2-sided 1-sample Wilcoxon signed rank test, α = 0.05, null: μ = 0, see Fig. 5 [a vs a]) and valence (p < 0.001, 2-sided 1-sample Wilcoxon signed rank test, α = 0.05, null: μ = 0, see Fig. 5 [v vs v]). Moreover, within group similarities of affect properties (a vs a; v vs v) are significantly different from between group (a vs v) similarities (p < 0.001, 2-sample Wilcoxon rank sum test, α = 0.05, Bonferroni corrected for multiple comparisons, null: μ_1_ = μ_2_). However, similarities between the affect properties are not significantly different from orthogonality (p = 0.31, 2-sided 1-sample Wilcoxon signed rank test, α = 0.05, null: μ = 0, see Fig. 5 [a vs v]).

## Discussion

At the core of the research findings presented here is the idea that a state of the human brain emerges in perceiving an affective stimulus. This brain state encodes the properties of the stimuli and is central to both the perception of affect as well as its elicited action tendencies reflecting the perception’s value, here modeled via SCR. To our knowledge, this is the first time the neural processing correlates of affective valence and arousal have been simultaneously characterized on a continuous scale in combination with a secondary validation measurement of affective reactivity.

The functional neuroanatomical encodings of affect perception that were extracted by our modeling methodology largely agree with neuroanatomical localizations of affect processing identified within both the univariate literature as well as a past MVPA-based classification study of the dimensional properties of affect^19^. A notable exception to this overlap is our finding of an encoding of positive valence in the superior parietal lobule, which is associated with visuospatial processing and attention^32^ and working memory^33^ rather than affect processing. One important difference between this work relative to a past MVPA-based classification study, which utilized similar brain state extraction methodology^19^, is the presence of male, female, and couple erotica in the current stimulus imageset. Past work by Bühler *et al*.^34^ found increased activation of the superior parietal sulcus in response to erotic imagery, suggesting a potential source of this difference.

We also identified voxels within brain regions related to the encoding of affective arousal and valence, specifically vlPFC (high arousal, neg. valence), right dlPFC (high arousal, neg. valence), pre-SMA (high arousal, neg. valence), and posterior insula (low arousal) that deviate from canonical affect processing brain maps identified both in univariate and multivariate approaches, but are (with the exception of posterior insula) well-known within the cognitive control literature and are implicated in volitional emotion regulation^35,36^. In light of other recent work^37^, which found that combining discrete and dimensional representations of emotion best characterizes the affective content of stimuli (in comparison to individual models), our findings support a hypothesis that one role of dimensional arousal may be to serve as a graded translation signal linking core affect and preparatory/modulatory responses to threats having multiple, discrete categorizations.

During the course of validating the neural encoding patterns identified via MVPA regression of perceived affect, we found that the neural encoding of valence is, in fact, orthogonal to the neural encoding of arousal. This implies that the neural activations related to positive and negative valence stimuli are independent from the neural activations related to high- and low-arousal stimuli, a core principle embedded into the circumplex model of affect^21^. This finding is at odds with earlier evidence of piece-wise linear correlations between these quantities in the positively and negatively valent half-planes^23^. A potential explanation for our study’s ability to unmask this orthogonality of major affective signals, an important finding which has gone unobserved in the literature, is the stimulus imageset that we selected for this experiment (see Materials and Methods: Image Stimuli Selection and Presentation). The imageset was chosen to maximize its span of the valence and arousal normative score subspace rather than replicate the density of the IAPS imageset’s underlying distribution in this space. In short, prior empirical evidence for the correlation of these measures may represent an artifact of the specific imageset’s sampling bias rather than its reflection of the true distribution of affective stimuli available. To our knowledge, this is the first time that cognitive measures have been explored using a similarity analysis of the Haufe-transformed encoding hyperplane, which we believe provides unique insight that is both qualitatively relevant and quantitatively verifiable.

Finally, given the strength and ubiquity of predictive effects demonstrated by the brain state model of affective processing in this work, we believe serious consideration should be given to long-term replacement of psychophysiological measurements of affect (specifically SCR) with fMRI MVPA-based measurements when and where it is methodologically and economically feasible to do so. As evidenced by this study, secondary psychophysiological measurements can be inferior to well-designed fMRI-based affect processing protocols.

Despite the success of our methodological approach in relating affective processes to fMRI BOLD brain state, we achieved, statistically, only medium effect-sizes (Pearson’s r), according to the canonical taxonomy^38^. If brain state is truly the central unit of affect processing then we must account for unexplained variance. We attribute the primary source of variance within our experiment to our use of normative affective scores averaged over the ratings of individuals of both sexes as well as variance contributions from other potentially important but hidden variables such as sexual orientation, age, education, and occupation. Indeed, past work has shown that normative assignments of valence and arousal scores reflect both individual variation^39^ as well as sex-dependent differences^40^. This explanation is further supported by the relatively strong correlations we observed for our inter-subject (i.e., group mean intra-subject) predictions of normative affect scores, which suggest that individual variations in the intra-subject models contribute to unexplained variance.

A second important source of variance is our measurement technology, fMRI. Due to its low-temporal fidelity, fMRI volumes capture only averaged neural activity within each spatially distinct region (voxel), rather than the truly causal neural activation patterns underlying brain dynamics. Thus, our use of the term brain state to describe fMRI-derived patterns of activation comports with the strict engineering definition of the term only in the context of low temporal fidelity. It is possible that spatial details of high-frequency activation patterns are lost and comprise the missing effect (therefore, the observed effect is comprised of commonly activated spatial regions). There exists indirect evidence to support this claim. Recent work using magnetoencephalography (MEG) to image the brain responses to visual emotional stimuli suggests that affective signal properties have unique temporal processing signatures^41^ operating on the scale of 100–300 msec, an order of magnitude less than could be detected by the standard fMRI methodology utilized in this work.

A further notable weakness of this study is that we did not simultaneously collect self-rating or psychophysiological measurement of the valence property of each of the 90 selected IAPS images. Independent behavioral measurements of the valence property of dimensional affect have been reported in the literature using facial electromyography (EMG)^23^ in which the zygomaticus (smile) muscle activity is correlated with positively valent stimuli and corrugator supercilii (frown) activity is correlated with negatively valent stimuli. fMRI-compatible EMG technology has also been validated using visual stimuli^42^. Placing brain state as the central unit of affect processing requires that independent measures of both valence and arousal are simultaneously modeled across the broadest set of emotion response types. Therefore, adding predictors such as EMG response to the common brain state-predicted modalities (subjective-experience via both self-report and normative scoring as well as autonomic nervous measurement via SCR) is clearly warranted.

This work provides support for the power of brain state to encode the multidimensional properties of affective stimuli. To address a critical limitation of this work, we are currently acquiring a larger set of data in which independent psychophysiological measurements of both valence and arousal are simultaneously captured along with fMRI BOLD signal. Beyond the heuristic value of this work to the emerging understanding of brain-affect relationship, we propose that the study’s methodology has potential benefits for informing a broad swath of fMRI-based research that relates to emotion processing and regulation. A stand-alone image presentation task that incorporates the imageset used in this work to fit our affect prediction models would consume only 584 s (9.7 m) of scan time if it was constructed using our task design parameters. By combining this task with their own image presentation paradigms, researchers could extract, via post-hoc analysis, measurements of perceived affective arousal that are more accurate than could be achieved through direct observation of fMRI-compatible SCR. Instructions for a minimum time variant of the design used in this work, which we term the Fast Affect Induction Design (FAID), including stimulus timing instructions and IAPS image IDs, is available in the Supplemental Materials.

## Conclusion

Our results demonstrate that functional brain states derived from fMRI BOLD beta-series encode both the perceived affective properties embedded within affective stimuli as well as their corollary SCR. Indeed, regression models of perceived affective arousal based on brain states exhibit effect sizes more than three times larger than those based on SCR, the psychophysiological response with which it is canonically associated. Further, the distributed neural activations that comprise these predictive brain states largely agree with past MVPA-based neural pattern classifiers of the valence and arousal properties of perceived affect as well as a growing body of literature of functional neuroanatomical representations of affect, suggesting that the underlying brain states are robust, generalizable, and predictive affective representations.

## Materials and Methods

### Overview

We analyzed data acquired from the Intrinsic Neuromodulation of Core Affect (INCA) study, an fMRI-based investigation of emotion perception, unguided emotion regulation, and real-time fMRI-guided emotion regulation. This study was conducted in the Brain Imaging Research Center (BIRC) at the University of Arkansas for Medical Sciences (UAMS). All participants provided written informed consent. All procedures were conducted with approval and oversight by the UAMS Institutional Review Board and were performed in accordance with the relevant guidelines and regulations.

The study’s procedures were conducted over two sessions, each scheduled on separate days. Session 1 was the assessment in which we obtained written informed consent, determined if participants met clinical exclusionary criteria, and administered all behavioral surveys and questionnaires. Session 2 was the neuroimaging session. Detailed descriptions of the study design, participant demographics, and acquisition equipment as well as MR and psychophysiological data preprocessing have been previously described by Bush *et al*.^7^ For clarity, we summarize here those important methodological steps that are necessary for appropriate comprehension and interpretation of our findings. The methods described for multivariate regression and significance testing of anatomical encodings are unique to this work.

### Participants

Our analysis included data from (n = 19) demographically diverse participants: age [mean (s.d.)]: 28.2 (9.2); sex: 10 (52.6%) female; race/ethnicity: 16 (84.2%) self-reporting as White or Caucasian, 2 (10.5%) as Black or African-American, 1 (5.3%) as Hispanic or Latino. All participants were right-handed, native-born United States citizens (which controls for potential cultural confounds in the IAPS normative scores), medically healthy, having no current psychopathology or current usage of psychotropic medication. All participants produced a negative urine screen for drugs of abuse immediately prior to the MRI scan. If necessary, participants’ vision was corrected to 20/20 during the MRI scan. Color-blindness was exclusionary.

### Image Stimuli Selection and Presentation

Our analysis pertains only to data acquired during the INCA experiment’s System Identification Task, the first of three tasks performed during Session 2 (for details see^7^). Within this task, our analysis focuses on the extrinsic image presentation formats, comprised of image stimuli (n = 90) that were drawn from the full IAPS imageset^39^ according to a maximum separation heuristic^7^ which insured that the selected image subset exhibited maximum inter-image Euclidean distances in the subspace of normative valence (v) and arousal (a) scores. Normative affect coordinates of the sampled images are depicted in Fig. 1, panel A. Images were presented for 2 s (stimulation) succeeded by a fixation cross for an inter-trial interval (ITI) sampled uniformly randomly from the range of 2–6 s. Stimulus times were balanced across two 9.25 min scans in order to minimize correlations between the theoretical regressors formed by convolving stimulus times with the canonical hemodynamic response function. These correlations were calculated between above and below average normatively scored stimuli, respectively, for valence and arousal.

### MR Image Acquisition, Preprocessing, and Beta-series Construction

Imaging data were acquired with a Philips 3 T Achieva X-series MRI scanner (Philips Healthcare, Eindhoven, The Netherlands). Anatomic images were acquired with a MPRAGE sequence (matrix = 256 × 256, 220 sagittal slices, TR/TE/FA = 8.0844/3.7010/8°, final resolution = 0.94 × 0.94 × 1 mm^3^. Functional images were acquired using a 32-channel head coil with the following EPI sequence parameters: TR/TE/FA = 2000 ms/30 ms/90°, FOV = 240 × 240 mm, matrix = 80 × 80, 37 oblique slices, ascending sequential slice acquisition, slice thickness = 2.5 mm with 0.5 mm gap, final resolution 3.0 × 3.0 × 3.0 mm^3^. MRI data preprocessing was conducted using AFNI (Version AFNI_16.3.20)^43^ unless otherwise noted. Anatomic and functional data underwent preprocessing according to pipelines and gray matter (GM) masking procedures previously detailed in Bush *et al*.^7^ To conduct MVPA we extracted features according to the beta-series method^44^. To summarize this method, we constructed the general linear model (GLM) of the image stimuli for two concatenated scans of the System Identification Task according to AFNI’s 3dDeconvolve function, making use of the individual modulation flag, -stim_times_IM, and the BLOCK4(2,1) model of the hemodynamic response function. The GLM was solved via AFNI’s 3dlss function, controlling for drift artifacts via the –polort A and –concat flags and controlling for motion artifacts by re-including a 24-dimensional motion model^45,46^. This process resulted in a series of whole-brain activation maps, one map for each image stimulus. These maps were then subsequently compressed to low-dimension according to Gram-Schmidt orthogonalization^47^.

### Skin Conductance Response (SCR) Acquisition, Preprocessing, and Beta-Series Construction

We recorded the psychophysiological measure of skin conductance via a BIOPAC MP150 Data Acquisition System (BIOPAC Systems Inc., Goleta, CA) in combination with the AcqKnowledge software platform and the EDA100C-MRI module. Electrodes were applied to each participant’s left hand; see Bush *et al*.^7^ for placement details. SCR signal was filtered according to a previously validated processing pipeline^48–50^, which involved median filtering, zero-centering, band-pass filtering, down sampling, and z-scoring^7^. Note, a first-order, unidirectional Butterworth band-pass filter (5 Hz low pass and 0.033 Hz high pass) was applied in this analysis rather than the bi-directional filter used in previous work. Also note that half of the SCR data for three subjects (1 of 2 runs each) was excluded from analysis (comprising 7.9% of the total SCR data) due to data corruption or the absence of a measurable SCR. These exclusions fall far below typical exclusions rates observed in studies involving SCR measurements^51^. From this data, we extracted SCR betas-series via a custom-implemented Matlab^52^ pipeline, which applied the same processing steps to the SCR design function as were applied to the raw SCR signal (to control for peak-shifting). The beta-series was then solved via Matlab’s regstats function.

### Multivariate (i.e., multivoxel) Pattern Analysis (MVPA)

We performed MVPA via linear support vector machine (SVM) regression^53^ using the Matlab Statistics Toolbox’s default implementation, fitrsvm (default parameters available on-line), to make fMRI-derived beta-series based predictions of the image stimuli’s normative affective scores and induced SCRs. For simplicity, our prediction experiments may be organized according to a previously published convention^7^, y_i_,_j_ = f(β_i,j_^P^), where f denotes the trained regression model (see section Intra-subject Regression Training and Cross-validation, below), y_i,j_ is the quantity to be predicted for the i^th^ subject and j^th^ stimulus; β^P^_i,j_ is the P-set of betas, P ∈ {GM,GS,SCR}, for the i^th^ subject and j^th^ stimulus, where GM denotes BOLD signal betas for gray matter voxels, GS denotes BOLD signal betas for Gram-Schmidt dimensions, and SCR denotes the SCR-derived betas (scalar values). Using this convention, we can parsimoniously describe the complete set of prediction experiments conducted in this study. Note, ~ y_i,j_(·) is the actual prediction for the j^th^ stimulus of the i^th^ subject based upon beta-series features (·):

$$\begin{array}{c} \sim {{\rm{v}}}_{{\rm{i}},{\rm{j}}}({\rm{GS}})={\rm{f}}({{{\rm{\beta }}}_{{\rm{i}},{\rm{j}}}}^{{\rm{GS}}}),\\  \sim {{\rm{v}}}_{i,j}({\rm{SCR}})={\rm{f}}({{{\rm{\beta }}}_{{\rm{i}},{\rm{j}}}}^{{\rm{SCR}}})\\  \sim {{\rm{a}}}_{{\rm{i}},{\rm{j}}}({\rm{GS}})={\rm{f}}({{{\rm{\beta }}}_{{\rm{i}},{\rm{j}}}}^{{\rm{GS}}}),\\  \sim {{\rm{a}}}_{{\rm{i}},{\rm{j}}}({\rm{SCR}})={\rm{f}}({{{\rm{\beta }}}_{{\rm{i}},{\rm{j}}}}^{{\rm{SCR}}}),\,{\rm{and}}\\  \sim {{{\rm{\beta }}}_{{\rm{i}},{\rm{j}}}}^{{\rm{SCR}}}({\rm{GS}})={\rm{f}}({{{\rm{\beta }}}_{{\rm{i}},{\rm{j}}}}^{{\rm{GS}}}).\end{array}$$

Further, we performed the following predictions in order to compare GM- versus GS-derived beta-series:

$$\begin{array}{c} \sim {{\rm{v}}}_{{\rm{i}},{\rm{j}}}({\rm{GM}})={\rm{f}}({{{\rm{\beta }}}_{{\rm{i}},{\rm{j}}}}^{{\rm{GM}}}),\\  \sim {{\rm{a}}}_{{\rm{i}},{\rm{j}}}({\rm{GM}})={\rm{f}}({{{\rm{\beta }}}_{{\rm{i}},{\rm{j}}}}^{{\rm{GM}}}),\,{\rm{and}}\\  \sim {{{\rm{\beta }}}_{{\rm{i}},{\rm{j}}}}^{{\rm{SCR}}}({\rm{GM}})={\rm{f}}({{{\rm{\beta }}}_{{\rm{i}},{\rm{j}}}}^{{\rm{GM}}}).\end{array}$$

### Intra-subject Regression Training and Cross-validation

MVPA regression prediction was cross-validated according to intra-subject leave-one-out cross-validation (LOOCV). That is, for the i^th^ subject, the j^th^ prediction was made using an SVM regression model trained on the set, T, of label-feature pairings [y_i,T_, β_i,T_^P^], such that T does not include j.

### Inter-subject Predictions

In support of our anatomical analysis of the group-wise distributions of intra-subject affect and SCR encoding parameters, we conducted inter-subject predictions of affect and SCR as follows. We predicted the normative valence score, normative arousal score, and SCR beta for the i^th^ subject and j^th^ image stimulus by averaging the predictions of the intra-subject fit hyperplanes for all k disjoint subjects (n = 18) applied to the j^th^ beta map of the i^th^ subject’s beta-series. These maps were aligned to the k^th^ subject’s hyperplanes according to the k^th^ subject’s gray-matter mask. We then modeled the normative affective scores and observed individual SCRs as functions of the group mean predicted measurements via GLMM. Random slope and intercept effects were modeled subject-wise. Effect-sizes (Pearson’s r) were calculated based on the resultant fixed-effects.

### Construction of Neuroanatomical Encoding Parameters

Linear SVM implementation of MVPA allowed us to generate anatomically relevant interpretations of how the brain encodes perceived affect in voxel activation space by converting the SVM decoding coefficients into their equivalent voxel encoding patterns via the Haufe-transform^14,16^. Encoding parameters were z-scored subject-wise.

### Neuroanatomical Encoding Parameter Significance via Permutation Testing

Due to the dependencies between voxel-wise encoding parameters dictated by the linear structure of the SVM regression hyperplane, we adapted permutation testing methods developed originally for searchlight-based MVPA decoding models^54^ to our single global (whole-brain) linear MVPA encoding models in order evaluate the group-level significance of the neuroanatomical encoding parameters, separately for valence, arousal, and SCR. The null hypotheses for our models assume that the labels of our affective stimuli contain no predictable information that may be encoded at a group level in the resultant brain states. Therefore, for valence and arousal, we formed the distribution of null voxel-wise encoding parameters by performing 1,200 random samples of the regression modeling process as follows. For each sample, the normative affect labels were randomly permuted. For each subject’s brain states, we then fit a linear SVM regression model as described in Materials and Methods: Multivariate Pattern Analysis. We then Haufe-transformed the resulting hyperplane parameters as described in Materials and Methods: Construction of Neuroanatomical Encoding Parameters. When then computed the group-level mean encoding and saved these parameters.

Following sampling, for each voxel falling within the group-level GM mask, we determined the fraction, f, of sampled parameters at that voxel location that are more extreme than the observed group-level mean parameter at that voxel location. If this fraction, f, is sufficiently small, f < 0.025 (2-sided test at p < 0.05), then the encoding parameter is deemed significant and reported. When testing SCR encoding parameters, for each permutation sample, individual subject’s SCR beta values were permuted according to a random order applied to all subjects of that sample. All other steps were identical to the affective permutation testing process.

### Measuring Effect Size via Mixed Effects Modeling

We measured the predictive effect size of the P-set of features (see Multivariate Pattern Analysis) via general linear mixed effects models (GLMM), in a manner similar to methods previously reported in Bush *et al*.^7^. In all effect size estimates, the measure of interest was the experimental measure and the predicted measure was the GLMM fixed-effect. Slope and intercept random effects were modeled subject-wise. We solved the GLMMs using Matlab’s fitlme function. Effect-size was calculated by computing the correlation (Pearson’s r) between the measure of interest and the predicted measure.

### Calculation of Cosine Similarity

Similarity is defined as the cos(θ) calculated as,

$$\cos ({\rm{\theta }})={\bf{a}}\cdot {\bf{b}}/({||{\bf{a}}||}_{2}{||{\bf{a}}||}_{2}),\ldots $$

where **a** and **b** are vectors and θ is the angle between them. The range of values of this similarity measure are [−1, 1] where a value of 1 denotes identical, −1 denotes opposite, and 0 denotes orthogonal.

## Electronic supplementary material

Supplemental Materials

## Data Availability

The datasets generated and analyzed during the current study are available from the corresponding author on reasonable request.
