# Supplementary material for: Common Functional Brain States Encode both Perceived Emotion and the Psychophysiological Response to Affective Stimuli
Source: Sci Rep. 2018 Oct 18;8:15444. doi: 10.1038/s41598-018-33621-6 (PMC6194055; doi:10.1038/s41598-018-33621-6)
Supplement: Supplementary file 1 — Supplemental Materials [file 41598_2018_33621_MOESM1_ESM.pdf]

Common Functional Brain States Encode both Perceived Emotion and the Psychophysiological Response to Affective Stimuli

Authors
Keith A. Bush, Anthony Privratsky, Jonathan Gardner, Melissa J. Zielinski, Clinton D. Kilts

Supplemental Materials

Supplemental Method: Fast Affect Induction Design (FAID)

By extracting out the extrinsic formats of the System Identification Phase of the INCA study design, we offer here a minimum time protocol for induction of perceived affect that would be expected to replicate the prediction results reported in this work. The 90 images of Stimulus Set A that were extracted from IAPS according to the maximum separation heuristic (see Materials and Methods: Image Stimuli Selection) are provided in the table below according to their IAPS IDs.

Extrinsic Format IAPS Image IDs
1750, 5982, 2540, 9120, 4641, 7100, 2520, 1050, 4503, 9832, 9360, 7175, 4619, 3310, 1022, 2020, 6300, 7211, 9331, 9622, 4531, 3250, 9254, 8475, 4626, 9435, 1931, 4800, 7043, 7285, 9163, 2279, 5750, 2058, 4770, 8160, 2722, 3500, 4550, 7490, 3015, 2205, 7031, 2351, 6930, 4598, 2040, 7492, 5950, 4597, 5010, 2095, 8158, 2217, 2352, 8190, 1301, 9415, 9184, 5760, 1620, 5833, 6550, 5395, 8200, 3102, 6563, 2222, 9220, 4490, 5020, 4235, 8186, 7217, 9102, 3000, 2271, 1333, 9426, 9700, 4649, 2302, 8231, 4220, 2795, 7480, 7224, 1810, 9390, 8030

Within the design, each image is presented for 2 s and stimuli are separated by an inter-trial interval (ITI) uniformly randomly sampled on the range 2–6 s. We also recommend an equilibration period prior to the start of image presentation of length 18 s as well as a post stimulus period of 30 s to ensure full acquisition of the tail of the HRF of the final stimulus presentation. Based on these timings, the full FAID requires [90*2 s = 180 s (stim)] + [89*4 s = 356 s (ITI)] + [18 s (equilibration)] + [30 s (post-stimulus)] = 584 s (9.7 m).

To generate the image stimulus ordering and timings we propose a modification to the selection process presented in this work (see Materials and Methods: System Identification Task) that includes only the following two constraints: 1) the FAID begins and ends with positively valent images; and, 2) four theoretical BOLD response models (v+, v-, a+, a-) should all be pairwise correlated less than 0.25. Note, these theoretical BOLD response models are constructed by first labeling each stimulus timing with a valence label (v+ or v-) and an arousal label (a+ or a-), which are based on the normative Likert score of the image linked to that stimulus timing (based on ordering) relative to the middle Likert score (5). The stimulus timings are then extracted based on label (i.e., forming image orderings and timings that include only a single affect property, e.g., v+) and convolving these timings with the HRF. Full experimental designs (image order and ITIs) should be re-sampled uniformly randomly until a simulated design simultaneously fulfills both criteria.

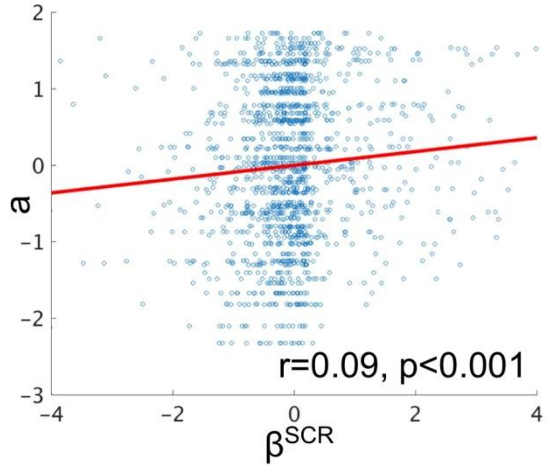

Supplemental Figure S1: *SCR predictions of the normative dimensional properties of arousal. GLMM of z-scored normative arousal as function of z-scored SCR state is significant (fixed effect: r=0.09, p<0.001, F-test). Circle markers indicate individual stimuli of the study. Red line depicts fixed-effect. Note, random slope and intercept effects that are near zero for all subjects are hidden by the plot of the fixed effect.*

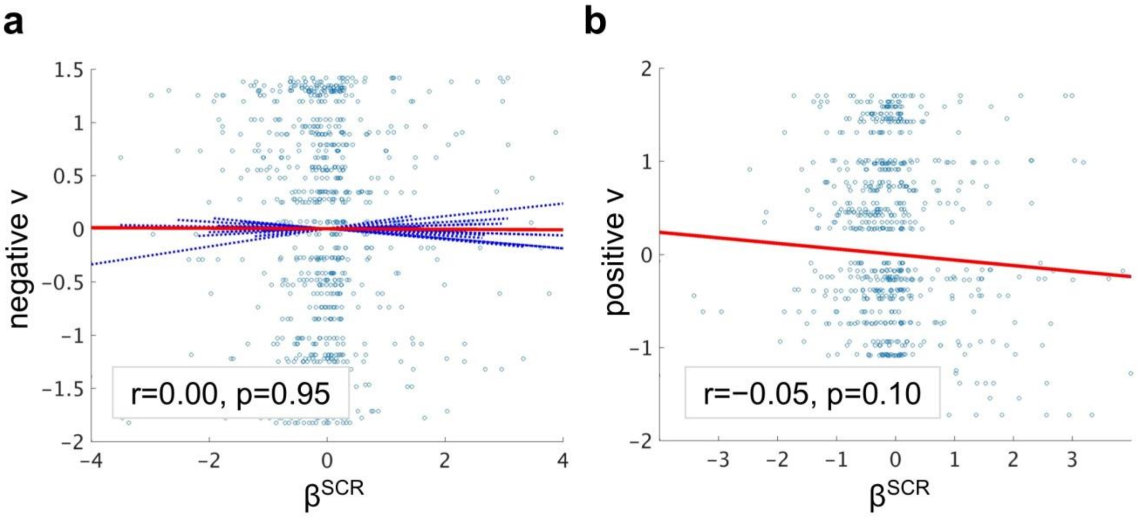

Supplemental Figure S2: *SCR predictions of the normative dimensional properties of valence.* ***(a)*** *GLMM prediction of z-scored normative negative valuence as a function of z-scored SCR state is not significant (fixed effect: r=0.00, p=0.95, F-test).* ***(b)*** *GLMM prediction of z-scored normative positive valence as a function of z-scored SCR state is significantly negative (fixed effect: r=-0.05, p=0.10, F-test). Circle markers indicate individual stimuli of the study. Red lines depict fixed-effects. Solid blue lines indicate significant random effects. Dashed blue lines indicated insignificant random effects. Note, random slope and intercept effects that are near zero for all participants are hidden by the plot of the fixed effect.*

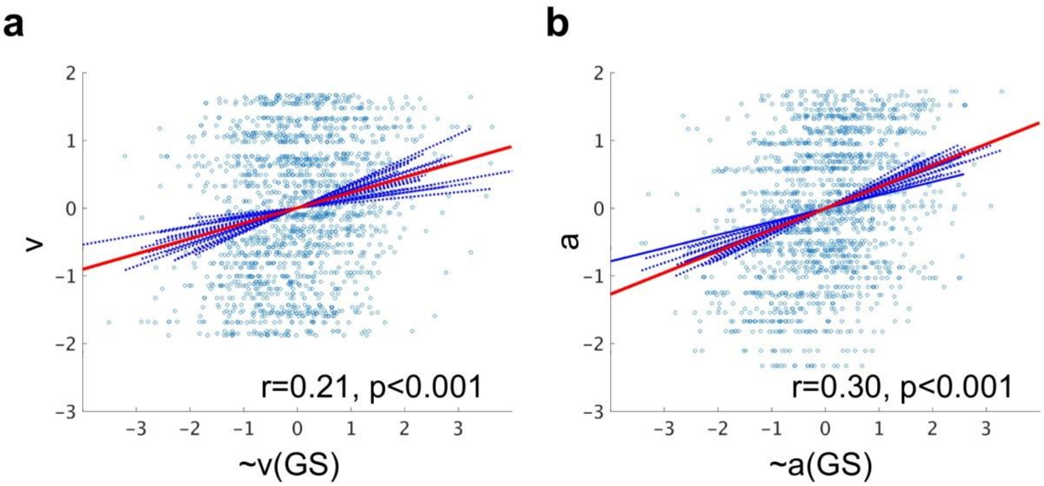

Supplemental Figure S3: *Affective brain state predictions of the normative dimensional properties of affect.* ***(a)*** *GLMM of z-scored normative valence as a function of the z-scored SVM-predicted normative valence, using Gram-Schmidt dimensionally reduced features as the model of brain state; SVM-predicted valence significantly predicts the true normative valence scores (fixed effect: r=0.21, p<0.001, F-test);* ***(b)*** *GLMM of z-scored normative arousal as a function of the z-scored SVM-predicted normative arousal, again, using Gram-Schmidt dimensionally reduced features; SVM-predicted arousal significantly predicts the true normative arousal scores (fixed effect: r=0.30, p<0.001, F-test). Circle markers indicate individual stimuli of the study. Red lines depict fixed-effects. Solid blue lines indicate significant random effects. Dashed blue lines indicated insignificant random effects.*

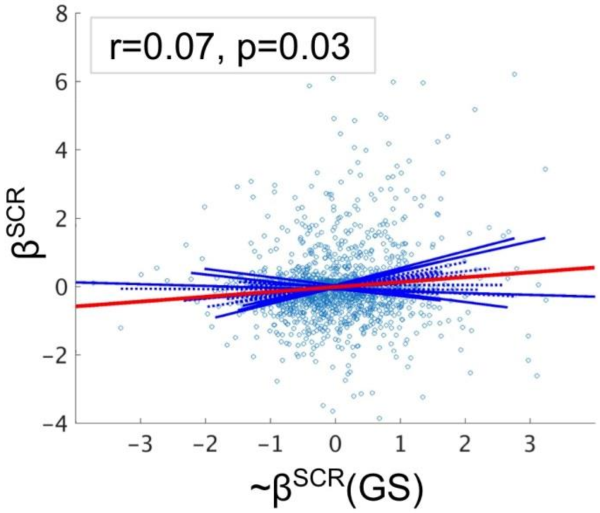

Supplemental Figure S4: *Affective brain state predictions of SCR state as captured by the beta-series method the normative dimensional properties of affect. SVM-predicted SCR state (z-scored) significantly predicts the z-scored measured SCR state (fixed effect: r=0.07, p=0.03, F-test). Circle markers indicate individual stimuli of the study. Red line depicts fixed-effect. Solid blue lines indicate significant random effects. Dashed blue lines indicated insignificant random effects.*

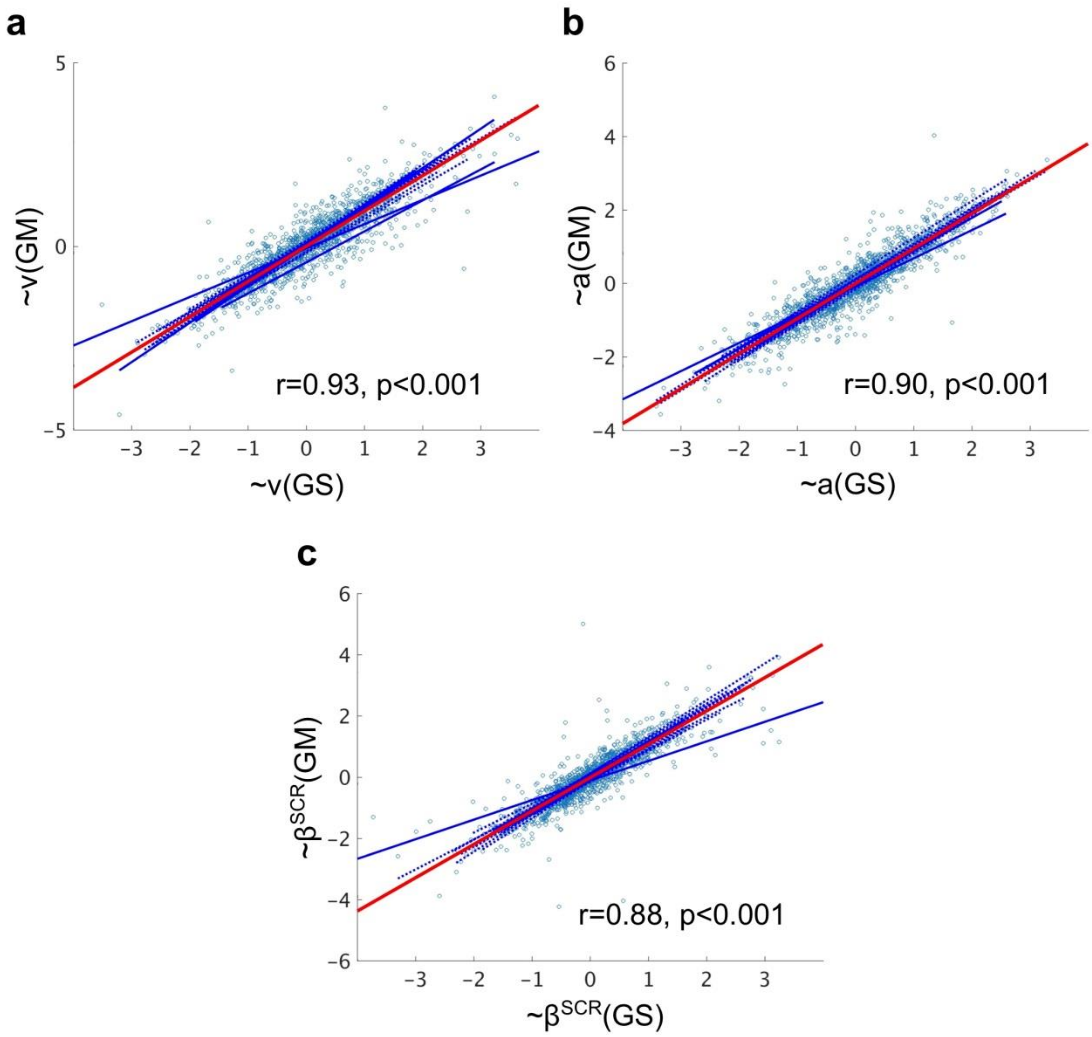

Supplemental Figure S5: *SVM predictions based on a Gram-Schmidt (GS) reduced dimensional features strongly predict SVM predictions based on whole-brain gray-matter (GM) features.* ***(a)*** *Valence predictions (fixed effect: $r=0.93$, $p<0.001$, F-test).* ***(b)*** *Arousal predictions (fixed effect: $r=0.90$, $p<0.001$, F-test).* ***(c)*** *Skin conductance response (fixed effect: $\beta=0.88$, $p<0.001$, F-test). Circle markers indicate individual stimuli of the study. Red lines depict fixed-effects. Solid blue lines indicate significant random effects. Dashed blue lines indicated insignificant random effects.*

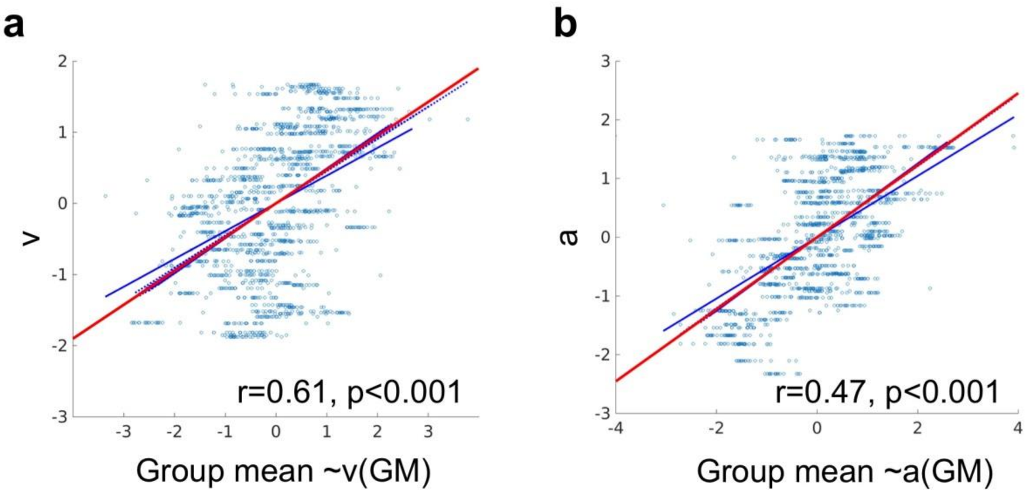

Supplemental Figure S6: *Inter-subject predictions (based on group mean intra-subject model predictions of hold-out gray matter (GM) beta-series features) strongly predict normative affect scores.* ***(a)*** *Valence predictions (fixed effect: r=0.63, p<0.001, F-test).* ***(b)*** *Arousal predictions (fixed effect: r=0.47, p<0.001, F-test). Circle markers indicate individual stimuli of the study. Red lines depict fixed-effects. Solid blue lines indicate significant random effects. Dashed blue lines indicated insignificant random effects.*

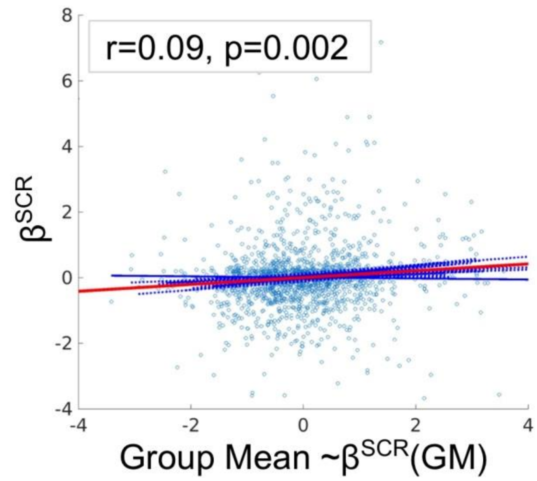

Supplemental Figure S7: *Inter-subject predictions (based on group mean intra-subject model predictions of hold-out gray matter (GM) beta-series features) significantly predict individual hold-out SCRs (fixed effect: r=0.09, p<0.001, F-test). Circle markers indicate individual stimuli of the study. Red lines depict fixed-effects. Solid blue lines indicate significant random effects. Dashed blue lines indicated insignificant random effects.*
